# Glucocorticoids-Induced Neuropsychiatric Disorders in Patients With Inflammatory Bowel Disease: A Systematic Review

**DOI:** 10.7759/cureus.28981

**Published:** 2022-09-09

**Authors:** Safina Ali, Salomi Paul, Shreyas Yakkali, Sneha Teresa Selvin, Sonu Thomas, Viktoriya Bikeyeva, Ahmed Abdullah, Aleksandra Radivojevic, Anas A Abu Jad, Anvesh Ravanavena, Chetna Ravindra, Emmanuelar O Igweonu-Nwakile, Pousette Hamid

**Affiliations:** 1 Medicine, California Institute of Behavioral Neurosciences & Psychology, Fairfield, USA; 2 Internal Medicine, California Institute of Behavioral Neurosciences & Psychology, Fairfield, USA; 3 Behavioral Neurosciences and Psychology, California Institute of Behavioral Neurosciences & Psychology, Fairfield, USA; 4 General Surgery, California Institute of Behavioral Neurosciences & Psychology, Fairfield, USA; 5 Neurology, California Institute of Behavioral Neurosciences & Psychology, Fairfield, USA

**Keywords:** inflammatory bowel disease, cognition, memory, depression, mood disorders, corticosteroids, prednisone, glucocorticoids

## Abstract

Inflammatory bowel disease (IBD) is a globally rising chronic intestinal disease that affects individuals in many parts of the world. Immunosuppressive medications such as corticosteroids are used to manage flare-ups and to induce remission in IBD. Corticosteroids are said to cause several systemic symptoms, but they are also associated with drug-induced neuropsychiatric disorders. This article examines the existing data on psychiatric and cognitive effects associated with corticosteroid therapy in relation to IBD. Many studies have found that corticosteroids appear to cause mood disturbances such as mania, hypomania, depression, and cognitive problems in the first few weeks of therapy, but these effects are dose-dependent and often mild. The purpose of this literature review is to shed light on the impact corticosteroids can have on individuals' mental health, which will aid physicians in the future when treating patients with IBD. Healthcare professionals should advise patients of this risk and assess the need for intervention. While there is evidence that corticosteroids can elicit neuropsychiatric symptoms, more data on people with IBD who are on corticosteroid therapy is needed to determine the prevalence of glucocorticoid-induced mood changes in this population.

## Introduction and background

**Figure 1:**
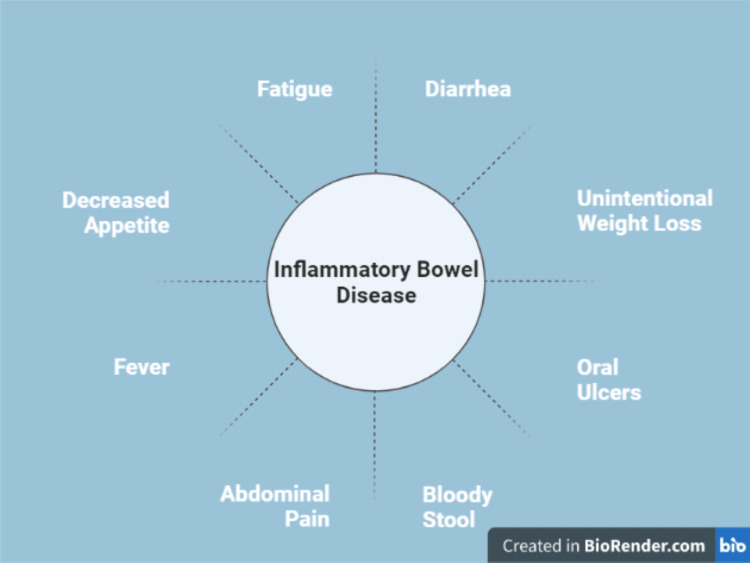
The symptoms of inflammatory bowel disease

Inflammatory bowel disease (IBD) is a highly prevalent chronic condition affecting around 6.8 million people worldwide [1]. It is characterized by chronic inflammation of the gastrointestinal tract. It includes ulcerative colitis and Crohn's disease. Symptoms include abdominal cramps, bloody diarrhea, weight loss, and fatigue [2]. Figure 1 describes the most common symptoms of IBD.

Crohn's disease is characterized by increased cytokine production, such as interleukin (IL)-12/IL-23 and interferons (IFNs)/IL-17, which affect the small bowel and colon, causing ulceration and full-thickness bowel wall inflammation, which frequently includes granulomas. Patients with Crohn's disease may also develop bowel strictures and fistulae. In contrast, ulcerative colitis is linked to higher IL-13 production, mainly affecting the colon, mostly involving the rectum, and spreading proximally. The symptoms are comparable to Crohn's disease, but it is not complicated by fistulae [2]. The prevalence and incidence of IBD are steadily rising, and the disease is becoming a global one. A recent systematic review published in 2017 discovered that the incidence of IBD is increasing in developing countries that are becoming more industrialized and Westernized, lending credence to the idea of IBD's global distribution [3]. Current medical therapy for IBD involves anti-inflammatory and immunosuppressive drugs such as corticosteroids, mesalamine compounds, azathioprine, thiopurine, anti-tumor necrosis factor (TNF) therapy, vedolizumab, tofacitinib, and many other agents [4,5].

Glucocorticoids are anti-inflammatory agents and immune modulators that are extremely effective in the treatment of a wide range of acute and chronic conditions, including immunological and inflammatory disorders like rheumatoid arthritis, systemic lupus erythematosus, Bechet’s disease, asthma, and inflammatory bowel disease [5,6].

Glucocorticoids are frequently used to treat flare-ups and induce remission in IBD, but they are not recommended as maintenance therapy due to a wide range of side effects [4-7,8]. Despite being extremely effective and useful, chronic glucocorticoid therapy has been linked to several systemic side effects, including osteoporosis, hyperglycemia, adrenal insufficiency, weight gain, hypertension, cataracts, impaired wound healing, and an increased risk of infections, as well as neuropsychiatric side effects such as depression, hypomania, and cognitive impairment [6,8,9]. 

This literature review aims to shed light on a variety of psychological disorders caused by chronic glucocorticoid therapy in the context of inflammatory bowel disease. Mood disorders are far more common in IBD patients, and chronic glucocorticoid therapy is one of the risk factors [8,10,11,12]. Patients should be educated about potential mood changes and encouraged to notify their healthcare providers; recognizing neuropsychiatric adverse outcomes will make it easier to deal with patients and recognize such occurrences sooner, which is crucial given that up to one-third of IBD patients may not adhere to treatment due to undesirable medication-related side effects. Early detection and awareness will facilitate communication between physicians and patients, potentially reducing non-adherence to therapy and contributing to the development of a strong patient-physician relationship, as well as improving the overall quality of life for these individuals [8]. 

## Review

Methodology

The Preferred Reporting Items for Systematic Reviews and Meta-Analyses (PRISMA) criteria were used to perform our systematic review [13].

Database

We started our research in March 2022, using a variety of online databases. We searched PubMed, PubMed Central, Medline, and Google Scholar to find clinically relevant observational studies, clinical trials, systematic reviews, and traditional reviews on glucocorticoid-induced neuropsychiatric disorders in inflammatory bowel diseases.

Research strategy

"Glucocorticoids", "prednisone", "corticosteroids," "mood disorders," "depression," "memory," "cognition," "inflammatory bowel diseases," and "intestinal diseases" were used as search terms.

Inclusion criteria

We only included peer-reviewed articles and studies published in English between 2001 and 2021. In the categories of systematic reviews, traditional reviews, observational studies, and randomized trials, we only included human studies. Various quality assessment tools were used to evaluate all the articles.

Exclusion criteria

Studies not in the English language and animal studies were excluded. We also excluded studies conducted before 2001.

Quality assessment tools

For systematic reviews and meta-analyses, we used the Assessment of Multiple Systematic Reviews (AMSTAR) questionnaire, the Cochrane risk-of-bias assessment tools for clinical trials, the New Castle-Ottawa questionnaire for observational studies, and the Scale for the Assessment of Narrative Review Articles (SANRA) for traditional reviews. We did not include studies that were of poor quality.

Data collection

Following quality control, data were collected individually from the final articles.

Results

**Figure 2:**
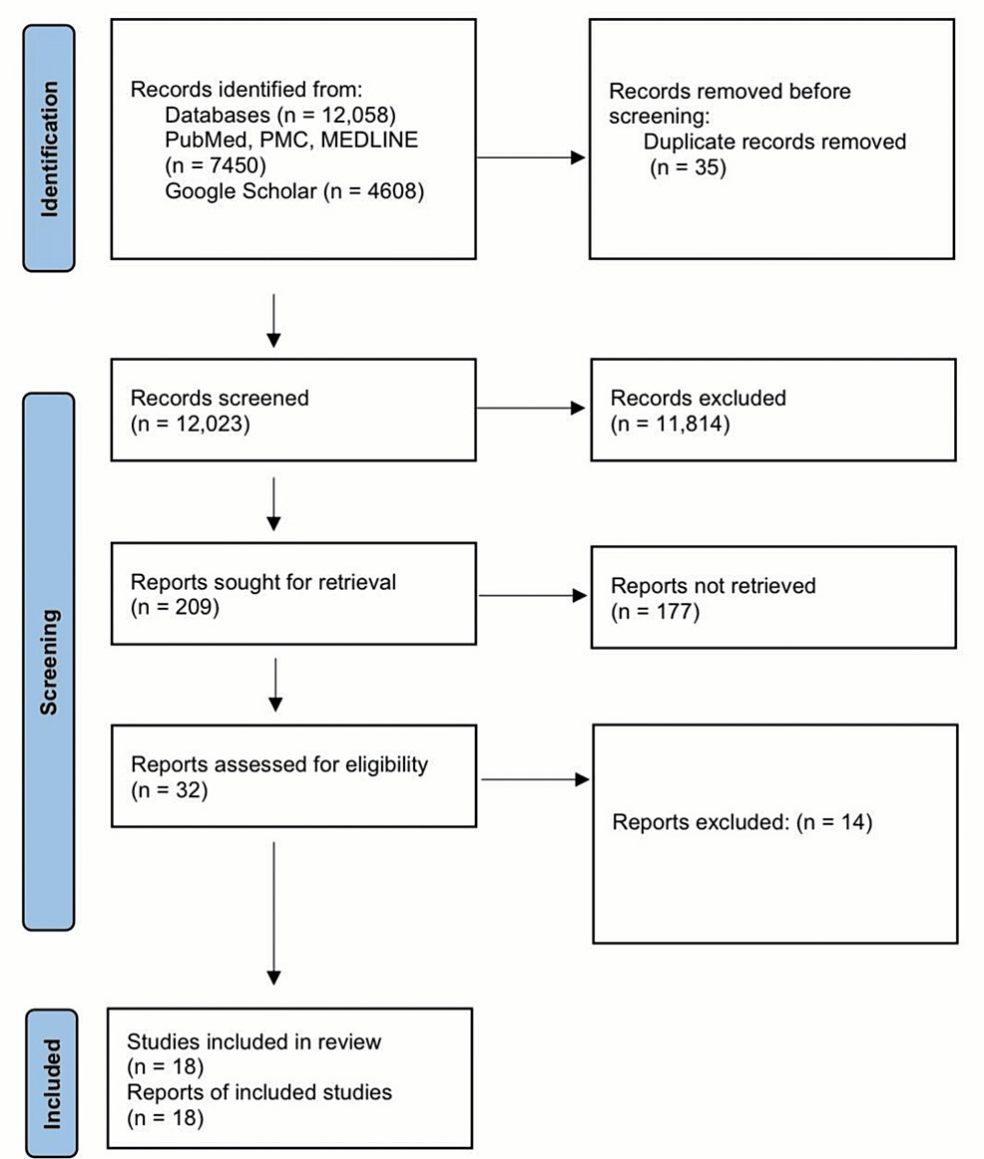
A PRISMA flowchart depicting the study selection process. PRISMA - Preferred Reporting Items for Systematic Reviews and Meta-Analyses

Medical Subject Headings (MeSH) keywords were used on PubMed, and regular keywords were used on Google Scholar, yielding a total of 12,058 articles. After we applied all the filters, we were left with 209 articles. We were able to collect 32 relevant articles for our review after screening these 209 articles including only free full-text articles and abstracts. These 32 articles were then evaluated using various quality assessment tools, and only 18 studies of high quality and relevance were chosen for our systematic review (Figure 2).

Discussion

Pathogenesis of Inflammatory Bowel Disease

**Figure 3:**
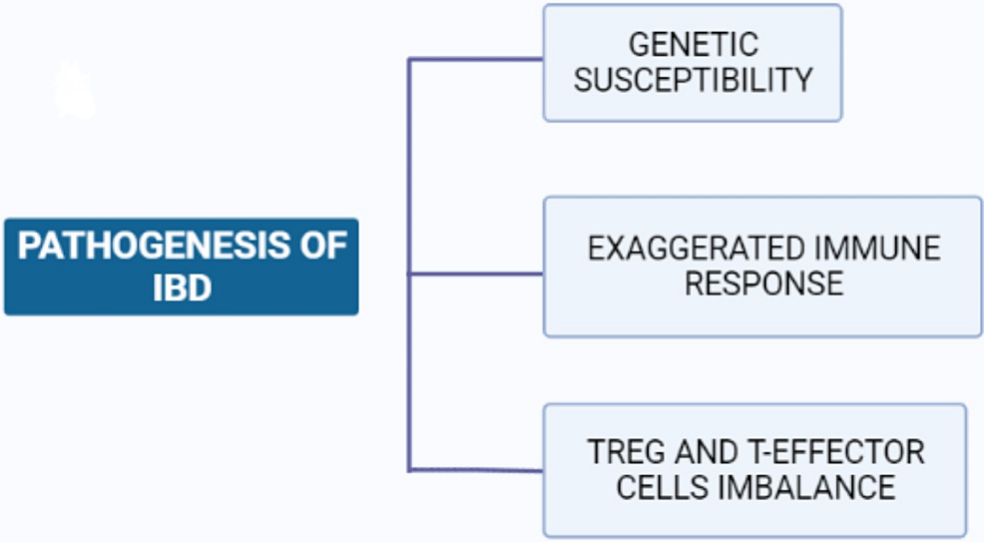
Pathogenesis of inflammatory bowel disease TREG CELLS: Regulatory T cells

Inflammatory bowel disease, which has a multifactorial etiology, is characterized by chronic inflammation of the gastrointestinal tract. A study in 2007 came up with two hypotheses regarding the pathogenesis of inflammatory bowel disease. The study concluded that inflammatory bowel disease results from an abnormal interaction between normal gut microbiota and the mucosal immune system, which results in an exaggerated immune response in genetically susceptible patients [2]. The second hypothesis proposes that abnormal mucosal immune responses in inflammatory bowel disease can be aided by factors such as changes in microbial composition and epithelial cell abnormalities. The condition is most likely caused by an imbalance between regulatory T cells (TREG cells) and effector T cells, which results in an excessive immune response that produces many pro-inflammatory cytokines, including IL-1, IL-6, IL-12p40, IL-23p19, tumor necrosis factor-alpha (TNFα), and interferon (IFN), which cause mucosal injury [2]. Figure 3 shows the pathogenesis of inflammatory bowel disease.

Incidence and Prevalence of Inflammatory Bowel Disease

The incidence and prevalence of IBD have been increasing worldwide. IBD is estimated to affect approximately 6.8 million people worldwide and has an age-standardized prevalence of 84.3 (79.2-89.9) cases per 100,000 people in 2017, with increasing incidence in developing countries that are becoming more Westernized [1].

Even though the incidence of IBD in advanced nations remains stagnant, the overall prevalence exceeds 0.3 percent. Europe and North America seemed to have the highest prevalence rates. In Europe, ulcerative colitis was 505 per 100,000 in Norway, while Chron's disease was 322 per 100,000 in Germany. In North America, ulcerative colitis was 286 per 100,000 in the United States, while Chron's disease was 319 per 100,000 in Canada. Table 1 shows the prevalence of IBD in Europe and North America.

**Table 1:** The prevalence of IBD in Europe and North America [3]

Country​	Prevalence of IBD​
Norway​	Ulcerative colitis 505 per 100 000​
Germany​	Crohn's disease 322 per 100 000 ​
The United States of America	Ulcerative colitis 286 per 100 000​
Canada​	Crohn's disease 319 per 100 000 ​

Since 1990, the incidence has increased in developing countries in Africa, Asia, and South America. In Brazil, the annual percentage change for Crohn's disease was +11·1% (95% CI 4·8-17·8) and the annual percentage change for ulcerative colitis was +14·9% (10·4-19·6). In Taiwan, the annual percentage change for Crohn's disease was +4·0% (1·0-7·1) and the annual percentage change for ulcerative colitis was +4·8% (1·8-8·0) [3].

Management of Inflammatory Bowel Disease

Many advances have been made in the treatment of IBD. The pathogenesis is complicated by an excessive immune system response. As a result, immunosuppressive medicines such as 5-ASA (Mesalazine), azathioprine, and corticosteroids are often used in the treatment of IBD patients [2,4,5].

Glucocorticoids (GCs) are strong anti-inflammatory and immunomodulatory medications that are used to treat a wide variety of acute and chronic inflammatory pathologies [8]. Glucocorticoids continue to be one of the most effective and first-line treatments for inducing remission in mild to moderate inflammatory bowel disease, but long-term therapy with corticosteroids is not recommended due to a variety of systemic side effects such as weight gain, hyperglycemia, cataracts, decreased bone mass density, mood disorders, and skin bruising. TNFα inhibitors such as infliximab and aminosalicylates are used in conjunction as steroid-sparing agents and in patients who do not respond to corticosteroids [2,4,5]. 

The Association Between the Development of Neuropsychiatric Symptoms and Corticosteroid Therapy

Long-term glucocorticoid therapy has many systemic side effects, but it also causes neuropsychiatric disorders. A review of the literature on mood and cognitive changes in corticosteroid therapy concluded that there are many psychological symptoms associated with chronic glucocorticoid therapy, including mania, hypomania, depression, and cognitive changes, specifical deficits in verbal or declarative memory. These symptoms are dose-dependent and resolve with therapy cessation. In this research study on mood and cognitive changes during systemic corticosteroid therapy, Brown et al reported that mood disturbances such as hypomania, mania, psychoses, and cognitive decline could manifest in individuals receiving steroids within two to three weeks. These neuropsychiatric symptoms were found to be dose-dependent. The risk of developing these symptoms is minimal (1.3%) if the daily corticosteroid dose is less than 40 mg; moderate if the dose is between 40 and 80 mg; and high (18.4%) if the dose exceeds 80 mg. Brown et al. also reported that low dose hydrocortisone (40 mg/day = 10 mg/day prednisolone equivalents) reduces the risk of cognitive impairment, whereas high dose hydrocortisone (160 mg/day = 40 mg/day prednisolone equivalents) increases the risk. The study also discovered a link between steroid medication and favorable psychological symptoms such as enthusiasm, being capable of thinking, and being extremely happy. These symptoms were mild and disappeared on their own when steroid therapy was stopped [9].

In another research study on "Mood changes during prednisone bursts in outpatients with asthma," Brown et al studied 60 asthma outpatients who were given prednisone bursts. Mood changes in these individuals were assessed using the Young Mania Rating Scale (YMRS) and activation subscale of the Internal State Scale (ISS) (clinician- and patient-rated measures of manic symptoms, respectively). Sixteen of the 32 people who came to the clinic for evaluation showed significant increases from normal 4.6 ± 1.4 days after starting prednisone therapy (mean initial dose: 41.9 ±7.8 mg/day). There were no significant increases in the Hamilton Rating Scale for Depression (HAMD). The study found that mood abnormalities such as mania and hypomania appeared within a week of beginning high-dose prednisone therapy (40 mg per day or more). Depressive symptoms did not appear as early in the therapy and are associated with prolonged exposure [14].

The Pathophysiology of Mood disorders in Glucocorticoid Therapy

The pathophysiology of steroid-induced psychosis is uncertain, and there’s limited data on the topic, however, synthetic steroids stimulate glucocorticoid receptors, interfering with the cortical pathway of the hypothalamic-pituitary-adrenal axis, resulting in mood problems [15]. In a literature review conducted by Judd et al., they elucidated the mechanism of neuropsychiatric disorders caused by synthetic corticosteroids. The hypothalamic-pituitary-adrenal axis regulates cortisol secretion in human bodies. The hypothalamus secretes cortisol-releasing hormone (CRH), which signals the pituitary gland to secrete adrenocorticotropic hormone (ACTH). ACTH then stimulates the zona fasciculata of the adrenal glands to release cortisol. Cortisol in our body acts through both glucocorticoid and mineralocorticoid receptors and plays a vital role in homeostasis. They came up with the hypothesis that exogenous glucocorticoids predominantly bind to glucocorticoid receptors over mineralocorticoid receptors in the body, which causes an extreme imbalance between glucocorticoids and mineralocorticoids and suppression of the hypothalamic-pituitary-adrenal axis, and that this may explain cognitive impairment and disturbed emotions experienced by many people during glucocorticoid therapy [16]. They also reported that increased resistance to cortisol and glucocorticoids was observed in individuals taking synthetic glucocorticoids, resulting in inhibition of brain-derived neurotrophic factor (BDNF). It is the low levels of BDNF that are thought to play a role in the development of anxiety and depression observed in these patients [16].

Prevalence of Mood Disorders in Inflammatory Bowel Disease

Depression and anxiety are common in people suffering from chronic conditions like IBD, and they have a negative impact on a patient's quality of life [7,10,11,17]. According to a study conducted by Nahon et al., in which they conducted a questionnaire for 1663 patients, 41 percent of people with IBD were anxious, and 11 percent were depressed. The study also indicated that participants with a history of smoking were more likely to suffer from anxiety, but not depression. Low socioeconomic status, flares, ongoing disease, and age were all risk factors for developing depression. Nahon et al. also found that a history of surgery was not related to increased depression [17].

Panara et al. reported in retrospective cohort research that neuropsychiatric disorders such as depression and anxiety are considerably more common in people with IBD than in the general population. The study also found a higher prevalence of depression in women with Crohn's disease than in males with Crohn's disease, although other studies observed no gender differences in depression rates [18]. This study discovered that an active disease was the most important risk factor, followed by gender and low socioeconomic status. Other variables that contribute to depression and anxiety in IBD patients include the negative effects and related mood disruptions of medications such as corticosteroids, biological agents, and immunomodulators, as well as the dread of side effects [17,18].

In a 2018 prospective observational study on the "Rate of corticosteroid-induced mood changes in patients with inflammatory bowel disease," 53 adult outpatients were given oral prednisone 40 mg for two weeks to see if there was a clinical association between corticosteroid use and mood changes. Patients were asked to complete questionnaires (the Beck Depression Inventory-II and the activation subscale of the Internal State Scale version two) before and two weeks after prednisone therapy [8]. To assess disease activity, the Harvey-Bradshaw Index and the Simple Clinical Colitis Activity Index were utilized. The study discovered that 49.1 percent of participants treated with oral prednisone for two weeks experienced mood alterations such as anxiety, agitation, and depression. The mood changes subsided after the steroid medication was stopped. There was no correlation discovered in this study between clinical disease activity and mood changes [8].

Treatment of Glucocorticoid-Induced Mood Changes

The recent data on the correlation between steroid therapy and neuropsychiatric disorders emphasize the need for physicians to be on the lookout for this impact, as it may go undiagnosed and patients may be hesitant to bring it to the knowledge of their providers. Dosage adjustment or cessation of steroid medication is usually the first step to improving drug-related mood changes, but it can take up to a few weeks to work, and it might be difficult to reduce or discontinue steroids in certain conditions that require steroid therapy.

Atypical antipsychotics like risperidone and olanzapine have also been implicated in the treatment of mood disturbances with glucocorticoid therapy and have shown a positive response. Studies have also suggested the use of lithium for corticosteroid-induced mania and depression, which have been shown to provide improvements in these symptoms [9,19]. Many anticonvulsants like carbamazepine, gabapentin, lamotrigine, and phenytoin have also been used for steroid-related psychiatric symptoms. For persistent steroid-induced psychosis, which fails to improve with other treatments, electroconvulsive therapy has provided significant improvement [19].

A case series on 20 patients who were on chronic glucocorticoid therapy and had steroid-induced psychiatric complications found that sodium valproate, which is a mood stabilizer, improved steroid-induced mania in these individuals [16,19].

Limitations

Our study had certain limitations. The majority of the data was collected from online databases such as PubMed and Google Scholar, and only free full-text articles and abstracts were included, so we may have overlooked some papers with more recent data. We also only included research published in English, and those published before 2001 were omitted.

## Conclusions

There are many literature reviews on the relationship between the use of corticosteroids and the development of neuropsychiatric symptoms. We noted that most studies had identified an increased prevalence of mood disorders and impaired cognition in individuals on glucocorticoid therapy. We found out that most neuropsychiatric disorders like mania, hypomania, and depression develop within the first few weeks after starting glucocorticoid therapy. These symptoms are dose-dependent, and the risk increases with higher doses. The symptoms are usually self-remitting and resolve upon discontinuation of glucocorticoid therapy.

We also found an increased prevalence of neuropsychiatric disorders such as depression and anxiety in people with inflammatory bowel disease. Many risk factors have been identified as contributing to the development of mood disorders in this population, including ongoing disease, a history of psychiatric illness, gender, low socioeconomic status, and high-dose corticosteroids. Although there is limited data on the relationship between corticosteroid use and mood disorders in IBD, many studies have shown that people on glucocorticoid therapy have a higher rate of mood disorders than the general population; however, more extensive research is needed to determine the role of corticosteroids in the development of mood disorders in this population. While inflammatory bowel disease is a severe condition with several systemic symptoms, physicians should also be aware of the neuropsychiatric symptoms that are common in these patients. Pharmacotherapy and cognitive behavioral therapy should be used to improve the overall well-being of these individuals.
